# Scintillating Micro-Probe Detectors for Conventional and FLASH Proton Dosimetry: Optical Transport and Geometry Modeling

**DOI:** 10.3390/bioengineering13101189

**Published:** 2026-10-13

**Authors:** Shiv K. Subedi, Sree Bash Chandra Debnath

**Affiliations:** Stritch School of Medicine, Loyola University Chicago, Maywood, IL 60153, USA

**Keywords:** inorganic scintillator detector, proton beam dosimetry, ultra-high dose rate, FLASH radiotherapy, optical Monte Carlo, scattering effects, light collection efficiency, GATE Monte Carlo simulations

## Abstract

Optical fiber-based inorganic scintillating detectors (ISDs) are increasingly investigated for radiation therapy applications, including microbeam and ultra-high-dose-rate dosimetry. Accurate characterization of these compact detectors is particularly important for proton beams delivered under high-flux and FLASH conditions. Simplified analytical and ray-tracing models can estimate detector saturation limits but may inadequately account for internal optical scattering and the fiber insertion geometry within the scintillating volume, leading to inaccurate estimates of the effective sensitive volume and dose-rate response. In this study, we developed a detailed computational framework that explicitly models the fiber–scintillator geometry and volumetric optical photon transport. Five detector geometries with different sensitive volumes were evaluated under 25 and 230 MeV proton irradiation using a fully volumetric GATE Monte Carlo optical transport model incorporating intra-dosimeter optical scattering. The results revealed a clear inverse relationship between detector sensitive volume and maximum measurable dose rate before saturation. At 25 MeV, the maximum measurable dose rate (${\dot{D}}_{\max}$) ranged from 364.5 ± 7.6 Gy/s for the smallest detector to 13.5 ± 0.2 Gy/s for the largest detector, while at 230 MeV, it ranged from 392.6 ± 8.1 Gy/s down to 15.6 ± 0.3 Gy/s at 230 MeV, respectively. These findings demonstrate that fiber insertion geometry and volumetric optical scattering significantly influence the dose-rate response and saturation threshold of scintillating micro-probes. Accurate modeling of these effects is therefore essential for optimizing detector geometry, establishing reliable quantitative dosimetry across conventional and proton-FLASH dose-rate regimes.

## 1. Introduction

Proton beam therapy (PBT) achieves superior spatial dose distribution compared to conventional photon therapy by taking advantage of the physical properties of the Bragg peak, thereby sparing adjacent healthy tissues [1,2,3,4,5]. Accurately resolving these steep dose gradients requires real-time beam monitoring systems equipped with sensitive volume detectors exhibiting high spatial resolution [6,7,8]. Furthermore, the clinical transition toward high-flux delivery systems and ultra-high dose rate (UHDR) paradigms, such as FLASH radiotherapy [9,10,11], introduces stringent operational constraints that real-time dosimeters must maintain a linear response under extreme photon generation rates before reaching signal saturation [12].

In proton dosimetry, both plastic scintillators and inorganic scintillator detectors (ISDs) are actively investigated, each offering distinct operational trade-offs. Plastic scintillators benefit from water-equivalent density and rapid temporal response, enabling direct dose rate measurements across scanned proton fields [13]. However, plastic scintillators exhibit pronounced non-linear ionization quenching near the Bragg peak due to high linear energy transfer (LET), alongside lower volumetric light yields that necessitate larger sensitive volumes to maintain adequate signal-to-noise ratios [13,14]. Moreover, a common issue for plastic scintillators is Cerenkov contamination due to the larger plastic volume that creates extra light under irradiation and requires significant corrections [7,15,16,17,18,19,20].

A wide range of fiber-coupled inorganic scintillators—including Gd_2_O_2_S:Tb/Eu/Pr, Y_3_Al_5_O_12_:Ce/Tb, Y_2_SiO_5_:Ce,Tb, CsI:Tl, ZnS:Ag, and (Zn,Cd)S:Ag—have previously been evaluated for radiation therapy and dosimetry applications for over two decades [21,22,23,24,25,26,27,28,29]. These materials exhibit emission spectra spanning the broad visible spectrum (from blue to red wavelengths) with decay times ranging from ns to μs. Comparative evaluations under low-energy radioactive environments (e.g., static beta sources) have shown varying relative scintillation efficiencies 8% for CsI:Tl, 5% for Y_3_Al_5_O_12_:Ce, and 15% for Gd_2_O_2_S:Tb relative to NaI) [22]. However, static benchmarks did not capture detector behavior in proton beam therapy (PBT). When evaluating fiber-coupled configurations in proton beams, it was found that while materials such as Gd_2_O_2_S:Tb, Y_3_Al_5_O_12_:Tb, and Y_2_SiO_5_:Ce,Tb suffer from significant ionization quenching, ZnS:Ag and (Zn,Cd)S:Ag yield superior performance due to strongly reduced quenching effects [21,30,31]. Recent works have also demonstrated the viability of fast red-emitting ISDs like ZnSe:O for time-resolved beam monitoring in brachytherapy and proton FLASH environments [32,33,34].

Building upon these developments, early comparative investigations established that silver-activated zinc cadmium sulfide—(Zn,Cd)S:Ag—embedded in polymer matrices provides exceptional volumetric light output and favorable response characteristics in proton energy ranges [8,21,35]. This heightened sensitivity allows for significant volume miniaturization down to sub-millimeter scales, making (Zn,Cd)S:Ag-based ISDs ideal for steep-gradient fields and accurate beam characterizations, provided internal light collection efficiency and count-rate saturation ceilings are accurately modeled.

However, fully realizing the potential of micro-volume detectors fabricated with ISDs requires accurate modeling of the internal light collection efficiency and bulk scattering within dense phosphor matrices. Existing theoretical models, however, rely on simplified ray-tracing assumptions that neglect bulk optical scattering and utilize a simple optical fiber insertion depth modeling with respect to the scintillator head [8]. The primary scientific novelty of this work lies in overcoming these physical approximations by introducing a realistic 3D model of the optical fiber depth within the scintillating volume and performing full Monte Carlo optical transport simulations that explicitly account for bulk light scattering.

Building on this approach, the primary objective of this study is to establish an accurate computational framework that combines precise detector boundary definitions with comprehensive optical transport modeling. Specifically, we aim to deliver precise dose-rate characterizations at two distinct energies—25 MeV and 230 MeV—to directly guide the design and optimization of micro-volume inorganic scintillators for both preclinical and clinical proton dosimetry applications.

## 2. Materials and Methods

### 2.1. Proton Beam Characteristics and Irradiation Setup

Proton irradiations were modeled based on the experimental beamline configuration at the Institut Pluridisciplinaire Hubert Curien (IPHC, CNRS, Strasbourg University). The facility utilizes a TR24 cyclotron (ACSI, Richmond, BC, Canada) delivering proton beams up to 25 MeV for passive radiobiology experiments [36]. The beam is scattered by an aluminum foil and collimated to establish a uniform lateral dose profile before extraction into air through a 5 mm-radius circular collimator defining the target field.

The irradiation environment was modeled as a broad, laterally uniform field encompassing the dosimeter volume. The primary proton fluence rate (*ϕ*) across the target cross-section is expressed as a function of the average beam current (*I*_beam_), elementary charge (*e*), and beam cross-sectional area (*A*_beam_):

(1) $$\phi =\frac{I_{\mathrm{beam}}}{e\cdot A_{\mathrm{beam}}}$$

where $A_{\mathrm{beam}}=\pi r_{\mathrm{col}}^{2}$, with $r_{\mathrm{col}}$ representing the radius of the circular collimator.

### 2.2. Inorganic Scintillating Detector Architecture

The single-photon scintillation detectors analyzed in this study are constructed according to established fabrication protocols [37,38,39]. Each sensor head consists of a composite matrix of inorganic scintillator grains—silver-activated zinc cadmium sulfide, (Zn,Cd)S:Ag (Phosphor Technology Ltd., Stevenage, UK). The scintillating mixture is dispersed within a polymethyl methacrylate (PMMA) binder at a 70:30 volume proportion. The scintillator exhibits a material density of 4.7 g/cm^3^ and a median particle diameter of 6.0 μm. This scintillating mixture forms an approximately ellipsoidal head attached directly to the cleaved end of optical fiber (105 μm core diameter, Thorlabs Inc., Newton, NJ, USA, FG105LVA).

**Figure 1:**
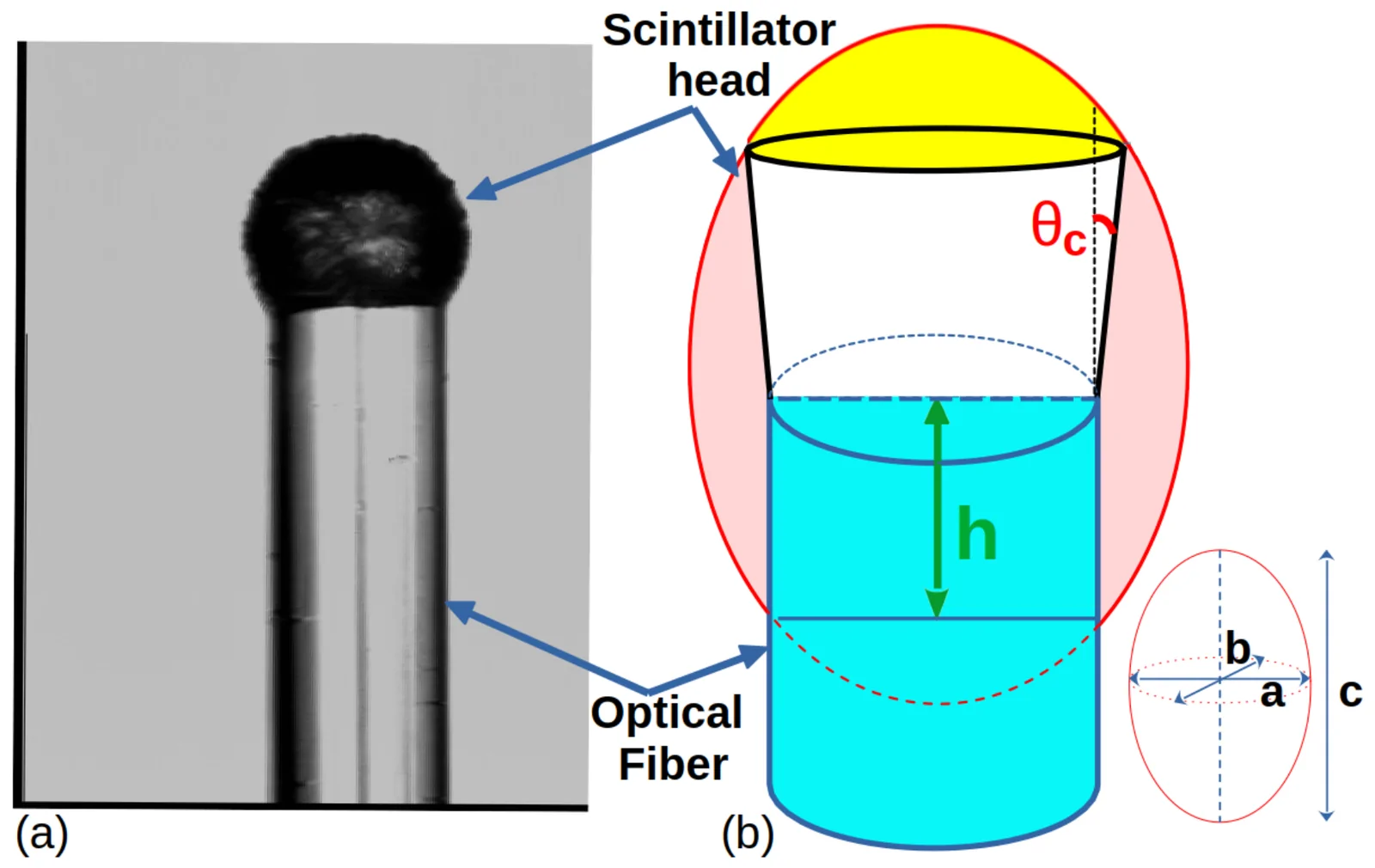
(**a**) Optical image of a typical lab-made fiber-based inorganic scintillator detector (ISD). (**b**) Schematic representation of the scintillator’s sensitive head.

**Figure 2:**
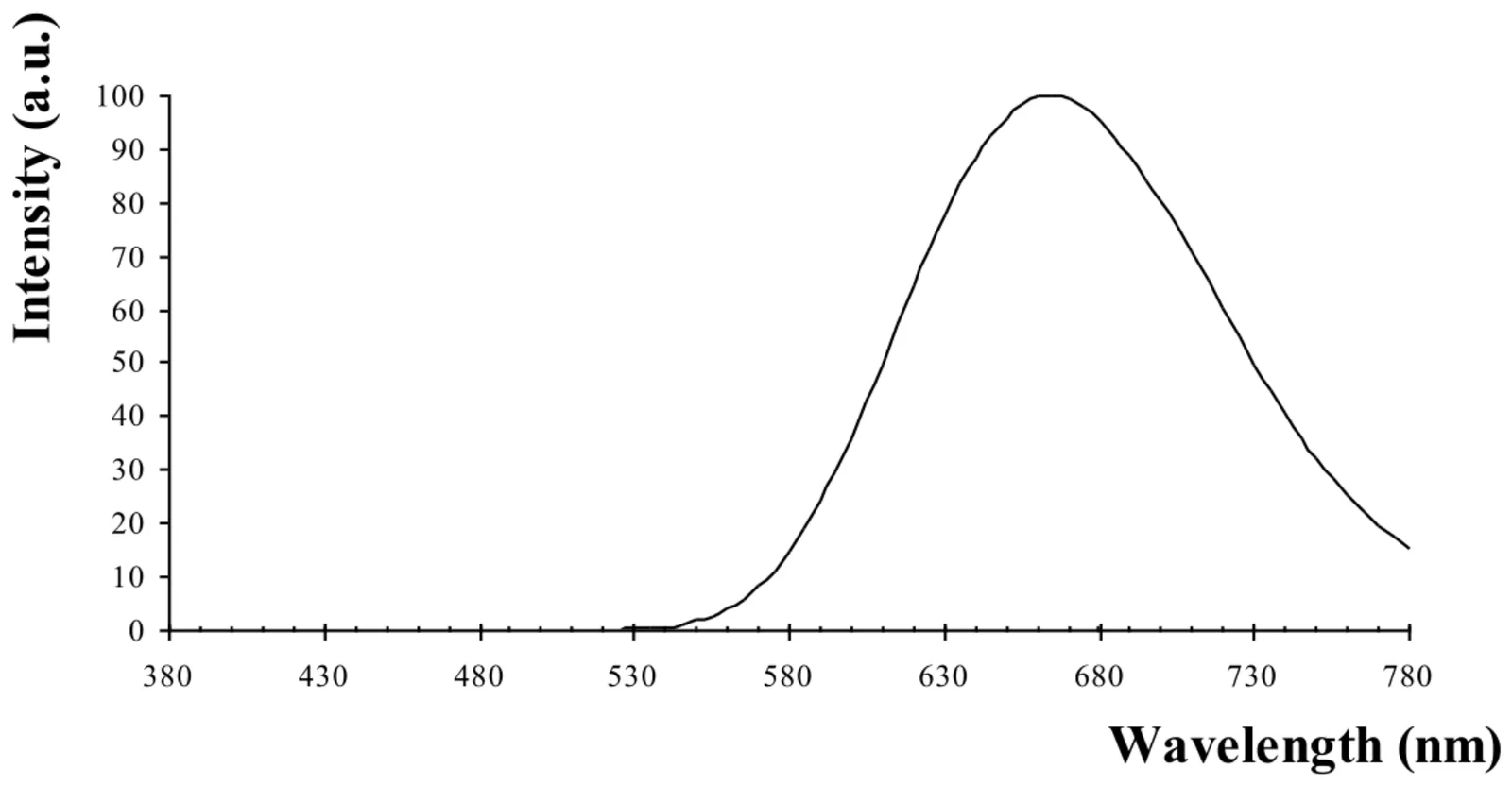
Normalized photoluminescence emission spectrum of (Zn,Cd)S:Ag scintillators, showing a peak emission at 660 nm. Figure is obtained from Phosphor Technology Ltd.

The physical dimensions of the detector’s active volume (ellipsoidal head geometry) are characterized by full-axes lengths *a*, *b*, and *c*, where the fiber insertion depth into the scintillating head is specified by *h*, as shown in Figure 1b. However, in the case where a=b=c, the ellipsoidal head of the scintillator reduces to a spherical shape. Primary proton energy deposition within the scintillator bulb generates isotropic scintillation light centered near *λ* = 660 nm (red emission), as shown in Figure 2.

A fraction of these emitted photons falls within the numerical aperture of the fiber core—defined by a critical acceptance angle (*θ*_*c*_) = 0.1 rad—establishing the effective optical collection domain within the scintillator head. Throughout this manuscript, this region is designated as the “effective volume” ($V_{\mathrm{eff}}$). For the ellipsoidal geometry, $V_{\mathrm{eff}}$ comprises two distinct geometric regions: a top truncated ellipsoidal cap (highlighted in yellow in Figure 1b) that transitions into a downward-tapering conical structure (highlighted in white in Figure 1b) extending toward the top of optical fiber interface.

### 2.3. Detection System Hardware and Readout Characteristics

Optical signal acquisition was performed using a single-photon counting module (SPD_1_VIS_M1, AUREA Technologies, Besançon, France) characterized by a 71% spectral quantum efficiency ($\epsilon_{\mathrm{quant}}$) and a maximum count-rate ceiling of 2 × 10^7^ photons/s. The photon counter follows a non-paralyzable response model with a dead time ($\tau_{c}$) of 20–40 ns. Measurements were recorded in continuous mode for 30 s at 1 s integration intervals.

### 2.4. Baseline Detection Framework and System Parameters

The single-photon counting channel response and dose-rate response model follow the dead-time characterization established by Simonin et al. [8].

For an incident proton beam characterized by fluence rate ϕ, the total rate of energy deposited (i.e., the incident energy flux) ($\phi_{E}$) within the efficient volume of the detector is determined by:  

(2) $$\phi_{E}=\phi \cdot S_{\det}\cdot E_{dep}$$

where $S_{\det}$ is the beam-projected cross-sectional surface area of the detector’s efficient volume and $E_{\mathrm{dep}}$ is the mean energy deposited per incident proton within the efficient volume.

**Figure 3:**
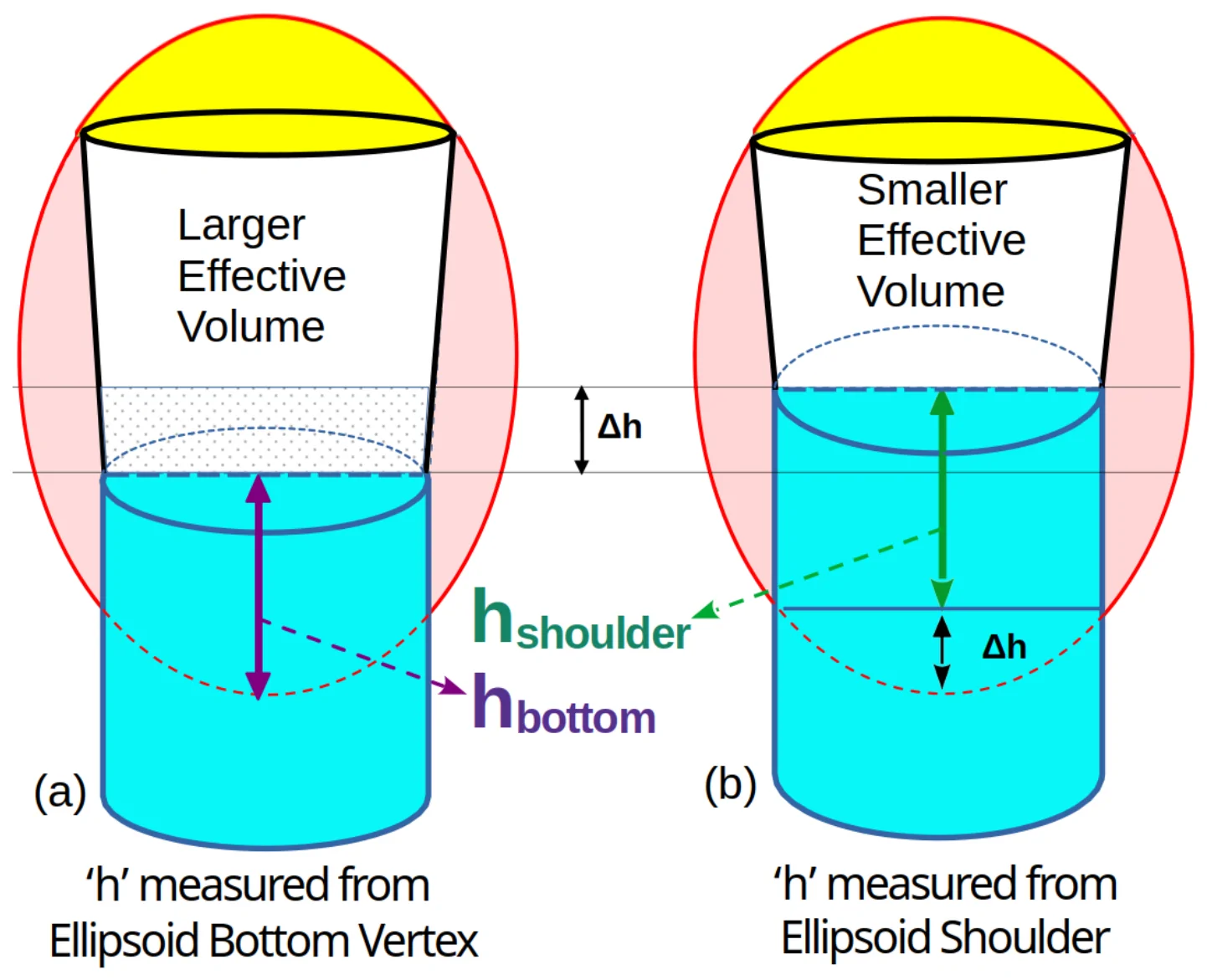
Schematic of the scintillator head illustrating the impact of fiber insertion length (h) measured from (**a**) the bottom vertex of the ellipsoid ($h_{\mathrm{bottom}}$) versus (**b**) the bottom shoulder ($h_{\mathrm{shoulder}}$) interface. The impact of the two modeling techniques is quantified by the parameter Δh.

The effective cross-sectional detection area ($S_{\det}$) is evaluated via numerical integration over the uninserted length of the scintillator head (c−h):

(3) $$S_{det}=2\int_{0}^{c-h}R_{eff}\left(x\right)dx$$

where x=0 corresponds to the fiber interface, ‘*c*’ is the total bulb length, and ‘*h*’ is the optical fiber insertion length within the scintillator head. As shown in Figure 3, when ‘*h*’ is measured from the bottom shoulder of the ellipsoid, $h\to h_{\mathrm{shoulder}}$. The local collection radius, $R_{\mathrm{eff}}\left(x\right)=\min\left[R_{\mathrm{core}}+x\tan\theta_{c},\;R_{\mathrm{scint}}\left(x\right)\right]$, models the expanding optical acceptance cone bounded by the fiber numerical aperture ($\theta_{c}=0.1\mathrm{rad}$) and the physical scintillator casing ($R_{\mathrm{scint}}$).

The photon arrival rate (i.e., the photon flux) incident on the readout module ($\phi_{\gamma ,\mathrm{counter}}$)—prior to electronic dead-time processing—is evaluated by accounting for the intrinsic light yield (*Y*), system optical efficiencies, and primary scintillator decay losses ($\tau_{s}$):

(4) $$\phi_{\gamma ,\mathrm{counter}}=\phi_{E}\cdot Y\cdot \epsilon_{\mathrm{geom}}\cdot \epsilon_{\mathrm{fiber}}\cdot \epsilon_{\mathrm{quant}}\cdot e^{-Y\phi_{E}\tau_{s}}$$

where $\epsilon_{\mathrm{geom}}$ is the optical light collection efficiency, $\epsilon_{\mathrm{fiber}}$ is the optical fiber transport efficiency, $\epsilon_{\mathrm{quant}}$ is the quantum efficiency of the photodetector, and $\tau_{s}$ is the primary luminescence decay time of the scintillator.

Accounting for the electronic processing dead time ($\tau_{c}$) of the acquisition system, the net output count rate recorded by the single-photon counter ($\phi_{\gamma }$) is expressed via the non-paralyzable dead-time model, as follows:


(5) $$\phi_{\gamma }=\frac{\phi_{\gamma ,\mathrm{counter}}}{1+\phi_{\gamma ,\mathrm{counter}}\cdot \tau_{c}}$$

By considering the maximum saturation throughput limit of the detection channel ($\phi_{\gamma ,\max}=2\times 10^{7}\;\mathrm{photons}/s$), the maximum detectable primary proton fluence rate ($\varphi_{\max}$) is determined. The corresponding maximum water-equivalent dose rate (${\dot{D}}_{\max}$) is evaluated directly using the linear energy transfer (LET) obtained from the NIST PSTAR database [40]:

(6) $${\dot{D}}_{\max}=\varphi_{\max}\cdot \left(\frac{\mathrm{LET}}{\rho_{\mathrm{water}}}\right)$$

where $\rho_{\mathrm{water}}$ is the mass density of water.

### 2.5. Methodological Refinements to Geometric and Optical Parameters

Recent studies evaluating the operational boundaries of inorganic scintillation dosimeters (ISDs) have developed generic framework models to calculate system dead times ($\tau_{c}$, $\tau_{s}$), macroscopic light yield (*Y*), and maximum detectable dose rates (${\dot{D}}_{\max}$) [8,35]. Despite providing valuable insights into non-paralyzable dead-time behavior, such formalisms exhibit two crucial structural and optical limitations.

First, conventional geometric modeling references the optical fiber insertion depth from the bottom vertex of the ellipsoidal bulb ($h_{\mathrm{bottom}}$) [8], whereas the core only becomes fully embedded at the shoulder interface ($h_{\mathrm{shoulder}}$), as shown by the offset parameter Δ*h* in Figure 3. For micro-probe dosimeters with dimensions comparable to the fiber scale, this conventional representation potentially affects the calculated detector cross-sectional area ($S_{\det}$), which can subsequently influence the accuracy of estimated mean energy deposited per incident particle ($E_{\mathrm{dep}}$). Second, previous estimates of light collection efficiency ($\epsilon_{\mathrm{geom}}$) relied solely on ray-tracing geometric acceptance, ignoring bulk optical scattering within the dense composite scintillator matrix—such as a 70% (Zn,Cd)S:Ag powder to 30% PMMA volume proportion—where multiple scattering may significantly alter photon path lengths and angular distributions prior to reaching the fiber core acceptance cone ($\theta_{c}$).

This study introduces a refined computational and analytical framework to resolve both limitations, as well as to guide correct geometrical model for maximum dose rate response. By redefining the fiber insertion reference plane at the shoulder interface and implementing full Monte Carlo optical transport simulations incorporating bulk scattering dynamics, parameters S_*det*_, E_*dep*_ and $\epsilon_{\mathrm{geom}}$ are evaluated. Consequently, the maximum detectable dose rate (${\dot{D}}_{\max}$) achieved by accurately modeled scintillating sensitive volume is reported.

### 2.6. GATE Monte Carlo Framework for Radiation and Optical Transport

Over the past several decades, the application of Monte Carlo (MC) methods in radiotherapy dosimetry has expanded significantly, establishing this simulation technique as a standard reference tool for complex radiation transport and detector modeling [41,42]. In this study, simulations were performed using GATE version 9.4.1 (built on the Geant4 toolkit, version 11.3.0), a simulation platform extensively validated and benchmarked for tracking radiation transport and optical photons in scintillation detectors across medical physics applications [43,44,45,46,47,48,49].

To accurately capture generated photons propagation and photon collection dynamics, GATE enables us to model bulk scattering, surface interactions, and fiber coupling dynamics. In this work, GATE Monte Carlo Simulations were primarily used to evaluate two key parameters: mean energy deposited per incident proton (E_*dep*_) within the effective scintillating volume ($V_{\mathrm{eff}}$) and geometric efficiency of the optical fiber ($\epsilon_{geom}$).

#### 2.6.1. Evaluating E_*dep*_

To evaluate $E_{\mathrm{dep}}$, monoenergetic proton beams were modeled under broad-beam uniform lateral illumination using the standard QGSP_BIC_HP_EMY physics constructor at the maximum deliverable cyclotron energy of 25 MeV and a clinically relevant FLASH energy of 230 MeV. Hits and energy deposition events occurring within the scintillator volume were accumulated across all primary proton trajectories $N_{p}$:

(7) $$E_{dep}=\frac{1}{N_{p}}\sum_{i=1}^{N_{p}}E_{\mathrm{dep},i}$$

where $E_{\mathrm{dep},i}$ is the total energy deposited by the *i*-th primary proton in the effective volume (V_*eff*_) and its associated secondary particles, as recorded in the ROOT output tree.

#### 2.6.2. Optical Simulations and Evaluating $\epsilon_{geom}$

**Figure 4:**
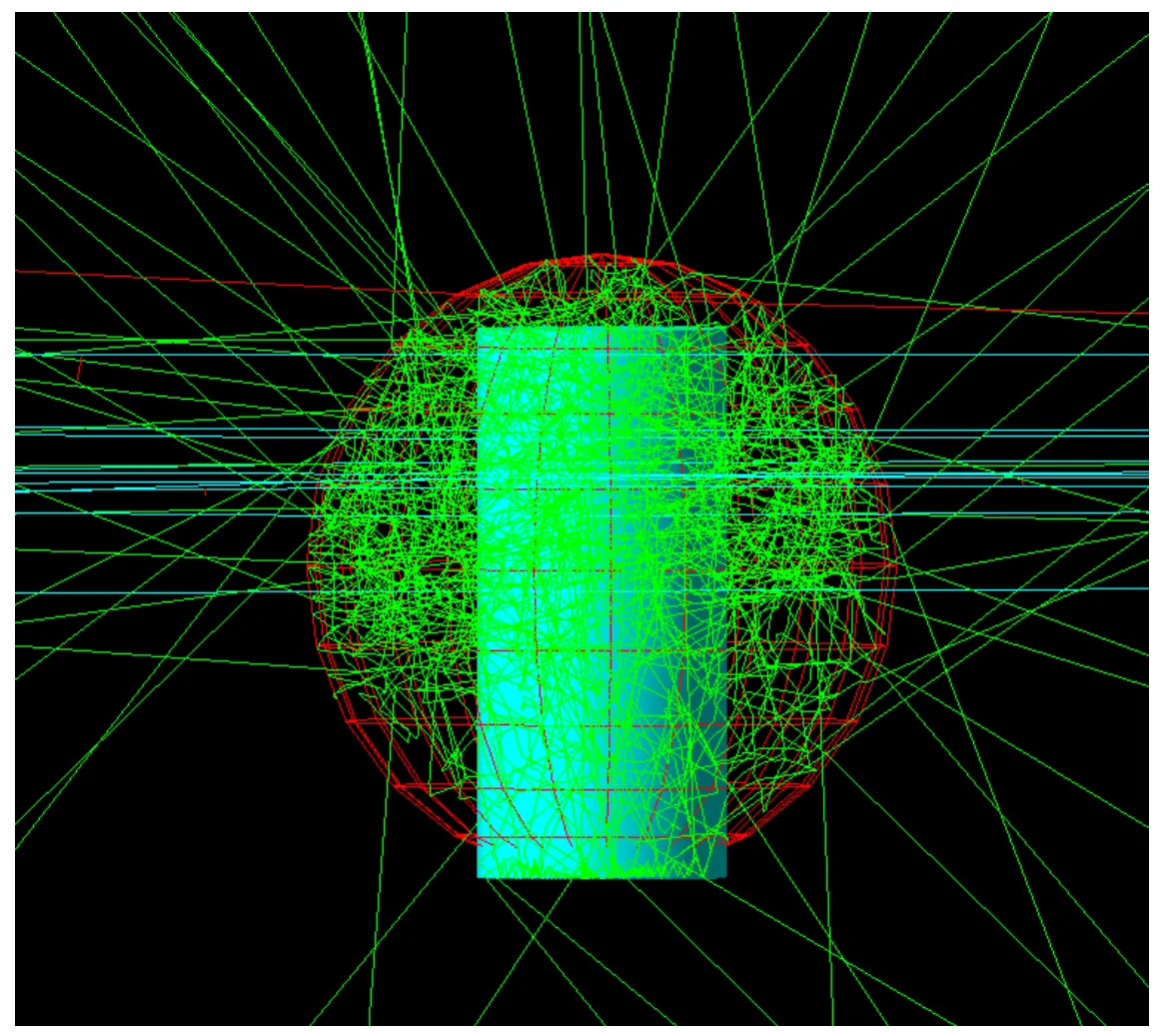
Visualization of optical photon transport and bulk scattering dynamics within the scintillator volume generated by 10 incident 25 MeV protons (reduced scintillation yield = 10 photons/MeV).

Unlike previous calculations [8], which evaluated geometric efficiency using a simplified ray-tracing approximation restricted strictly to optical photons within an idealized effective volume, the present work implements a fully volumetric optical transport framework. In this approach, optical photons were generated stochastically across the entire three-dimensional volume of the scintillator in GATE following the interaction of 25 MeV incident protons, accurately reproducing realistic energy deposition patterns rather than assuming localized or idealized emission (Figure 4).

The Monte Carlo model tracked individual photon trajectories through multiple internal scattering events within the powder–binder matrix, bulk volume attenuation, refraction at dielectric boundaries, and complex interactions at both the outer crystal surfaces and the optical fiber interface. Scintillation photons ($\lambda_{\mathrm{peak}}\approx 660\mathrm{nm}$, τ=10μs [50]) were generated isotropically using a reduced yield of 10 photons/MeV to optimize computational efficiency. Intra-dosimeter scattering was modeled using the Henyey–Greenstein Mie phase function [51,52] with a mean free path ($\lambda_{\mathrm{Mie}}=6.0\;\mu m$) matched to the median phosphor grain size ($D_{50}=6.0\;\mu m$) used in the scintillator detector.

At the outer boundary region of the top of optical fiber, photons were registered as accepted if their incident angle satisfied the fiber acceptance limit ($\theta_{c}=0.1\;\mathrm{rad}$). The baseline simulated geometric collection efficiency ($\epsilon_{\mathrm{geom},\mathrm{MC}}$) was evaluated as:

(8) $$\epsilon_{\mathrm{geom},\mathrm{MC}}=\frac{N_{\mathrm{accepted}}}{N_{\mathrm{generated}}}$$

where $N_{\mathrm{generated}}$ is the total number of distinct optical photons generated throughout the scintillator volume and $N_{\mathrm{accepted}}$ is the number of photons meeting the acceptance criteria at the fiber interface. From Equation (8), we see that $\epsilon_{\mathrm{geom},\mathrm{MC}}$ is strictly an intrinsic geometric property that tracks the fraction of total generated photons reaching the optical fiber tip. Because this propagation is governed solely by the emission wavelength and Mie scattering within the phosphor-binder matrix, $\epsilon_{\mathrm{geom},\mathrm{MC}}$ seems not dependent on the incident proton energy.

While volumetric Monte Carlo transport captures internal light scattering far more accurately than basic ray tracing, idealized Monte Carlo geometries assume uniform physical boundaries, uniform boundary layers, and strict Numerical Aperture cutoffs. They cannot fully account for physical micro-scale variations—such as boundary scattering from surface roughness, adhesive meniscus refraction, fiber alignment tolerances, trapped micro-air voids, and localized deviations from the nominal 70:30 scintillator-to-PMMA binder volume proportion. To bridge these simulation constraints and reflect physical probe construction, an optical coupling factor (*k*) was introduced to scale the baseline Monte Carlo geometric efficiency ($\epsilon_{\mathrm{geom},\mathrm{MC}}$) into the true geometric collection efficiency ($\epsilon_{\mathrm{geom}}=k\cdot \epsilon_{\mathrm{geom},\mathrm{MC}}$).

In principle, ‘*k*’ could be replaced by explicitly modeling surface roughness and air voids in Monte Carlo. However, doing this in practice would require detailed characterization of the actual detector microstructure and interfaces, for example, using micro-imaging (such as micro-CT) to characterize void distributions and surface/profilometry measurements to quantify interface roughness. These measurements could then be incorporated into the Geant4/GATE optical model through explicit geometries and/or appropriate optical boundary parameters [53], significantly increasing simulation complexity. A simpler physical model could eventually be used once these variations are better understood. Using the empirical factor ‘*k*’ is a straightforward, computationally efficient way to capture real-world manufacturing differences while preserving the underlying energy-deposition and scintillation physics of the model. For a single fabricated detector, these differences are assumed to remain unchanged with incident proton fluence. Therefore, a single value of ’*k*’ was used for each detector over the full investigated fluence range.

### 2.7. ISD Detector Geometries and Physical Specifications

**Figure 5:**
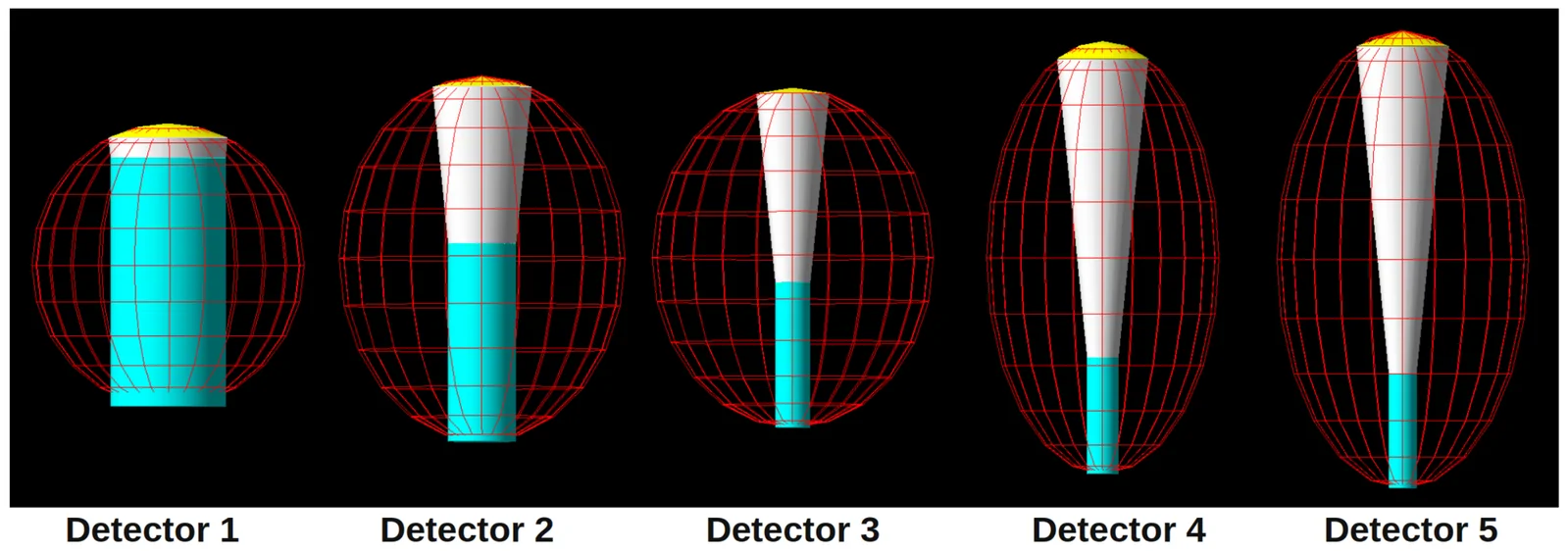
Geometric configurations of Detectors 1–5. Scintillator heads are shown in red, optical fiber in cyan, and the white and yellow regions together define the effective volume ($V_{\mathrm{eff}}$).

The general simulation and analytical framework described above was applied to five distinct inorganic scintillation detector geometries (designated as Detectors 1–5), as shown in Figure 5. These configurations span a broad range of sensitive volumes ($V_{\mathrm{eff}}$), optical fiber insertion length (h), and overall scintillation bulb dimensions (a, b, c), as presented in Table 1.

The physical detector configurations (Detectors 1–5) analyzed in this work were maintained identical to those characterized by Simonin et al. [8] to compare our results with respect to the real-detector’s geometric configuration achieved by using micrographic techniques. However, compared to the model in [8], the fiber insertion parameter ‘*h*’ is re-indexed relative to the detector’s ellipsoidal shoulder ($h_{\mathrm{shoulder}}$) rather than the bottom vertex ($h_{\mathrm{bottom}}$) to establish a physically practical insertion boundary.

In the experimental setup, the detectors are oriented for broad-beam lateral illumination and positioned centrally within the radiation field, a few millimeters downstream of the exit collimator.

## 3. Results

### 3.1. Detector Physical Parameters and Geometric Efficiency

The optical fiber insertion length discrepancy parameter (Δh), detector surface areas (*S*_det_ of the effective volume), mean energy depositions (*E*_dep_ within the effective volume), simulated geometric light collection efficiencies ($\epsilon_{\mathrm{geom},\mathrm{MC}}$ from Monte Carlo simulations), and the optical coupling factor (k) across all five detector configurations are summarized in Table 2. $E_{\mathrm{dep}}$ was evaluated for two proton beam energies of 25 MeV and 230 MeV, denoted as $E_{\mathrm{dep},25}$ and $E_{\mathrm{dep},230}$, respectively.

The parameters $S_{\det}$, $\epsilon_{\mathrm{geom},\mathrm{MC}}$, and $E_{\mathrm{dep}}$ have negligible uncertainty contributions across all evaluated detector configurations. Since the parameter $S_{\det}$ was computed via adaptive numerical integration of a cubic spline interpolated profile, it yielded negligible numerical uncertainty (<$10^{-10}$) [54]. $S_{\det}$ values are reported as rounded integers (precision ≤ 0.5 μm^2^) in Table 2. Statistical uncertainties for $\epsilon_{\mathrm{geom},\mathrm{MC}}$ remained ≤0.00056% across all detectors (evaluated via Poisson ratio propagation). Finally, statistical uncertainties on $E_{\mathrm{dep}}$—evaluated as the standard error of the mean ($\mathrm{SEM}=\mathrm{std}/\sqrt{N}$), where *N* is the total number of recorded events within the scintillating volume—remained below $5.5\times 10^{-4}\;\mathrm{MeV}$ (<0.55keV) across all configurations due to high simulation statistics of $10^{8}$ primary incident protons considered in this study. Because these associated uncertainties are negligible, explicit numerical error bounds for $S_{\det}$, $\epsilon_{\mathrm{geom},\mathrm{MC}}$, and $E_{\mathrm{dep}}$ were not included in Table 2 for visual clarity and excluded from the fitting procedure; consequently, they do not propagate into the estimation of ${\dot{D}}_{\max}$.

Defining the fiber insertion boundary depth (*h*) from the detector shoulder significantly altered the structural and energy parameters. The resulting depth shift (Δ*h*) contracts both the contact surface area ($S_{det}$) and the effective physical volume ($V_{eff}$), which ultimately suppresses the deposited energy ($E_{dep}$)—an effect that is most significant in the smallest configuration (Det 1). As the active volume increases from Det 1 to Det 5, $S_{\det}$ expands from 2956 μm^2^ to 300,466 μm^2^, driving a corresponding increase in mean energy deposition per proton pass ($E_{\mathrm{dep},25}$ ranging from 0.413 to 1.132 MeV, and $E_{\mathrm{dep},230}$ ranging from 0.070 to 0.182 MeV). In contrast, the optical simulation results show a continual decrease in geometric efficiency, falling from 0.281% for Det 1 down to 0.008% for Det 5.

### 3.2. Global System Calibration and Non-Linear Detector Response

To determine the global system parameters governing detection channel saturation, the geometric parameters ($S_{\det}$, $E_{\mathrm{dep}}$, and $\epsilon_{\mathrm{geom}}$) were integrated directly into the non-linear response model (Equations (1)–(4)). The rate of energy deposited per unit time ($\phi_{E}$) was calculated via Equation (2) using the calculated cross-sectional areas ($S_{\det}$) derived from the revised shoulder boundary condition ($h_{\mathrm{shoulder}}$).

**Figure 6:**
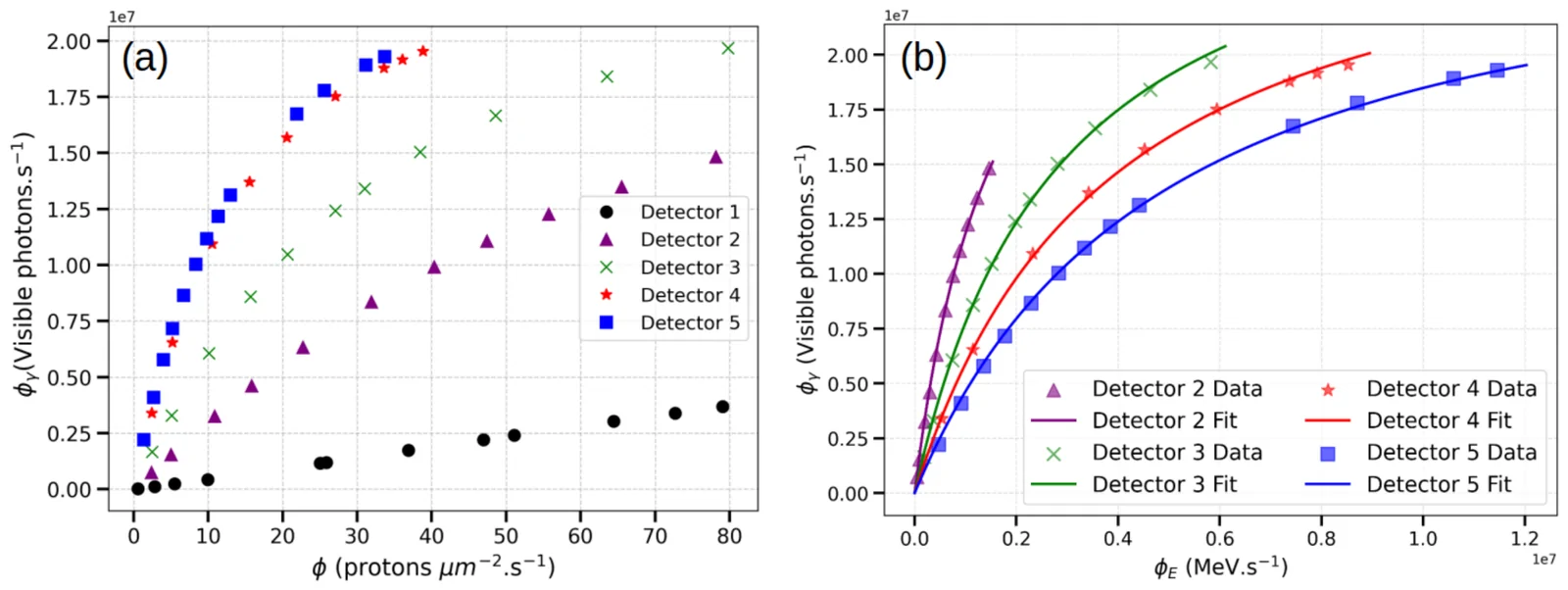
Detector’s photon flux (count-rate) saturation behavior: (**a**) Count rate ($\phi_{\gamma }$) versus proton fluence rate (ϕ) for Det 1–5; (**b**) Count rate ($\phi_{\gamma }$) versus energy deposition flux ($\phi_{E}$) for Det 2–5 showing the dead-time model fit from Equation (5).

Figure 6a displays the measured count rate ($\phi_{\gamma }$) across all five detectors (Det 1–5) as a function of primary proton fluence rate (ϕ). Figure 6b shows $\phi_{\gamma }$ against incident energy deposition flux ($\phi_{E}$), along with the non-linear model fits (Equation (5)). In the fitting procedure, optical fiber transmission was fixed at $\epsilon_{\mathrm{fiber}}=0.98$, evaluated from the manufacturer’s transmission spectrum weighted over the scintillator emission wavelengths. The quantum efficiency of the single photon counter was fixed at $\epsilon_{\mathrm{quant}}=0.71$. To ensure a consistent comparison across detector geometries and isolate geometry-dependent effects, the intrinsic material properties, including the scintillation light yield (*Y* = 16,072 photons/MeV) and luminescence decay time ($\tau_{s}=1.6\mathrm{ps}$), were held constant at their established values [8].

A shared global fit across Det 2–5 established a common electronic dead time of $\tau_{c}=30.79\pm 0.35\;\mathrm{ns}$, which was subsequently applied to cross-calibrate Det 1. Across all five detectors (Det 1–5), the extracted optical coupling factors spanned 1.34≤k≤6.20 (as shown in Table 2), resulting in true geometric efficiencies ($\epsilon_{\mathrm{geom}}$) ranging from 0.0496% to 0.3767%. All fits demonstrated excellent agreement with experimental counts across the full fluence rate dynamic range ($R^{2}\geq 0.9980$).

### 3.3. Maximum Dose Rate Evaluation

Using the parameters evaluated from the fitting procedure, the maximum detectable primary proton fluence rate ($\varphi_{\max}$) was determined at the saturation throughput limit of the detection channel ($\phi_{\gamma ,\max}=2\times 10^{7}\;\mathrm{photons}/s$). The corresponding maximum water-equivalent dose rate (${\dot{D}}_{\max}$) was then evaluated using Equation (6) for both 25 MeV and 230 MeV proton energies. Table 3 summarizes the maximum dose rates alongside the maximum fluence rates for detectors Det 1–5 for two proton energies of 25 MeV and 230 MeV.

## 4. Discussion

During proton interactions with the sensitive scintillating volume, evaluating the physical boundary conditions and optical dynamics provides a more accurate basis for dose rate calculations than earlier estimates [8]. Measuring the insertion depth ($h_{\mathrm{shoulder}}$) from the detector shoulder corrects the geometric baseline compared to previous work that measured from the ellipsoid base. This correction changes the effective cross-sectional area ($S_{\det}$) and average energy deposited per proton (E_*dep*_), with the largest relative impact occurring in the smallest detector (Det 1). In addition, replacing basic ray-tracing with optical Monte Carlo simulations accounts for internal photon scattering, yielding a more realistic geometric collection efficiency ($\epsilon_{\mathrm{geom}}$). To bridge simplified Monte Carlo transport models ($\epsilon_{\mathrm{geom},\mathrm{MC}}$) and practical light scattering dynamics inside the detector, introducing the optical coupling factor (*k*) accounts for real-world interface dynamics, such as boundary scattering from surface roughness, optical adhesive meniscus index matching, trapped micro-air voids, and manufacturing alignment tolerances. These structural and optical updates resolve earlier underestimations and establish a more reliable input baseline for overall detector throughput. From Figure 6b, it is observed that the dead-time model provides an excellent agreement with the experimental data ($R^{2}$ > 0.99) using a single constant ‘*k*’ value for each detectors across the entire range of the incident energy flux ($\phi_{E}$), which confirms that ‘*k*’ is not dependent on incident energy flux of the proton beam.

From a clinical perspective, these revised values define clear operational limits for fiber-integrated scintillation dosimeters in ultra-high dose rate (FLASH) radiotherapy. The results confirm a direct link between the detector’s geometrical configuration and saturation, i.e., the smallest volume (Det 1) achieves the highest maximum dose rate (${\dot{D}}_{\max,25}=364.5\pm 7.6\;\mathrm{Gy}/s$ at 25 MeV and ${\dot{D}}_{\max,230}=392.6\pm 8.1\;\mathrm{Gy}/s$ at 230 MeV), while larger detectors (Det 2–5) saturate at lower flux levels because they deposit more energy per proton ($E_{\mathrm{dep}}$) within their active volume, generating more scintillation photons per event. Across all detector geometries, ${\dot{D}}_{\max}$ evaluated at 230MeV was higher than at 25MeV. Because $E_{\mathrm{dep}}$ per proton is lower at 230MeV due to reduced stopping power, a higher incident primary proton fluence rate ($\phi_{\max}$) is required to reach the photon counter’s throughput saturation limit ($\phi_{\gamma ,\max}=2\times 10^{7}\;\mathrm{photons}/s$), directly translating to a higher maximum dose rate. For Det 1, these thresholds represent a notable improvement over the previously reported ${\dot{D}}_{\max}$ values of 295±2.6Gy/s at 25 MeV and 337±3.0Gy/s at 230 MeV [8]. These results suggest that smaller scintillator geometries provide a higher effective sensitive-volume fraction for photon interactions, resulting in enhanced response under high-dose-rate conditions. For FLASH beam monitoring above 40 Gy/s [55], reducing active volume is therefore essential to delay count-rate saturation in non-paralyzable readout channels.

This study has a few modeling limitations. First, the optical Monte Carlo simulations modeled light collection assuming direct coupling into the fiber core at the tip interface, omitting the full cladding profile and outer housing geometry at the fiber entry. Accounting for cladding dynamics at the fiber tip could further refine the geometric collection efficiency ($\epsilon_{\mathrm{geom}}$). Second, while the optical coupling factor (*k*) captures physical interface losses and volumetric variations (e.g., slight deviations from the nominal 70:30 scintillator-to-binder ratio across probe sizes), directly modeling micro-scale voids and surface roughness in future simulations could reduce reliance on empirical scaling factors. Third, dose rate calculations assumed continuous beam delivery, whereas clinical FLASH beams often use pulsed time structures that may alter instantaneous photon rates and dead-time behavior during peak pulses. Fourth, in this study, we did not separately evaluate the individual contributions of Cherenkov radiation and intrinsic fiber luminescence. Future work will investigate these distinct optical signal components under high dose rate conditions. Additionally, we will investigate variations in optical fiber geometry relative to the scintillator volume, alongside evaluating alternative scintillating materials, to identify optimal compact detector configurations, enabling miniaturization of the detector volume while enhancing dose-rate sensitivity under FLASH proton beam conditions.

## 5. Conclusions

This study evaluated the maximum dose rate (${\dot{D}}_{\max}$) capabilities for fiber-integrated inorganic scintillation detectors by considering key geometrical and optical parameters. Referencing optical fiber insertion depth from the physical fiber-scintillator interface provided a more accurate geometric baseline for the active volume and cross-sectional surface area. In addition, optical Monte Carlo simulations accounted for internal photon scattering, yielding a more realistic geometric collection efficiency, which is important for measuring accurate dose rate and dose rate capabilities.

Applying these parameters to a non-paralyzable dead-time model confirmed that dose rate saturation is strongly governed by detector’s volume, as larger sensitive volumes generate higher optical photon fluxes that saturate the readout channel at lower dose rates. These findings highlight clear design trade-offs: reducing active volume delays dose rate saturation, making smaller detectors suitable for ultra-high dose rate (FLASH) dosimetry, whereas larger volumes may remain useful for conventional, high-sensitivity applications at lower fluxes. However, shrinking the detector must be balanced against maintaining sufficient energy deposition per proton to preserve an acceptable signal-to-noise ratio. Overall, accurate modeling of these parameters enables optimization of detector geometry, signal-collection efficiency, thereby supporting reliable quantitative dosimetry across conventional and high-dose-rate proton beam conditions.

## Figures and Tables

**Table 1:** Detector specifications used in this project.

Detector	a (μm)	b (μm)	c (μm)	h (μm)
1	240	260	260	216
2	440	450	570	300
3	860	860	1040	440
4	840	740	1470	390
5	1070	870	1740	430

**Table 2:** Detector specifications for Detectors 1–5, including the fiber insertion length discrepancy parameter (Δ*h*), effective cross-sectional area ($S_{\det}$), Monte Carlo geometric efficiency ($\epsilon_{\mathrm{geom},\mathrm{MC}}$) and optical coupling factor (k) evaluated using the revised fiber insertion depth (h_*shoulder*_) and optical photon transport. The deposited energies within the effective volume for 25 MeV and 230 MeV proton beams are designated as $E_{\mathrm{dep},25}$ and $E_{\mathrm{dep},230}$, respectively.

Detector	Δh (μm)	S_*det*_ (μm^2^)	*E*_*dep*,25_ (MeV)	*E*_*dep*,230_ (MeV)	*ϵ_geom,MC_* (%)	k
1	12.21	2956	0.413	0.070	0.281	1.34 ± 0.01
2	8.01	33,475	0.559	0.093	0.077	2.22 ± 0.02
3	3.89	96,935	0.752	0.122	0.029	3.13 ± 0.03
4	6.52	222,080	0.988	0.161	0.012	5.50 ± 0.08
5	5.11	300,466	1.132	0.182	0.008	6.20 ± 0.06

**Table 3:** Maximum water-equivalent dose rates (${\dot{D}}_{\max}$) for detectors Det 1–5 evaluated at 25 MeV and 230 MeV.

Detector	${\dot{D}}_{\max,25}$ (Gy/s)	${\dot{D}}_{\max,230}$ (Gy/s)
1	364.5 ± 7.6	392.6 ± 8.1
2	54.6 ± 0.8	61.7 ± 0.9
3	27.5 ± 0.5	32.1 ± 0.6
4	14.0 ± 0.2	16.3 ± 0.2
5	13.5 ± 0.2	15.6 ± 0.3

## Data Availability

The data presented in this study are available upon request from the corresponding author.

## Abbreviations

The following abbreviations and symbols are used in this manuscript:

ISD	Fiber-integrated inorganic scintillator detector
FLASH	Ultra-high dose rate radiotherapy
${\dot{D}}_{\max}$	Maximum measurable dose rate
*h*	Fiber core insertion depth
$S_{\det}$	Apparent surface of detector efficient volume
$E_{dep}$	Mean energy deposited per proton
GATE	Geant4 Architecture for Medicine Applications
$\epsilon_{\mathrm{geom}}$	Geometric efficiency
$\epsilon_{\mathrm{geom},\mathrm{MC}}$	Baseline Monte Carlo geometric efficiency
*k*	Optical coupling factor
$\tau_{c}$	Photon counter dead time
PMMA	Poly(methyl methacrylate)
LET	Linear energy transfer
NA	Numerical aperture
$\theta_{c}$	Acceptance angle of the optical fiber
$D_{50}$	Median grain size of phosphor particles
$\lambda_{\mathrm{Mie}}$	Mie scattering mean free path
*Y*	Macroscopic light yield
$\epsilon_{\mathrm{fiber}}$	Optical fiber transmission efficiency
$\epsilon_{\mathrm{quant}}$	Photon counter quantum efficiency
$\tau_{s}$	Scintillator luminescence decay time
ϕ	Proton fluence rate
$\phi_{\gamma }$	Measured photon count rate
$\phi_{E}$	Incident energy deposition flux
